# Supplementary material for: Content-rich biological network constructed by mining PubMed abstracts
Source: BMC Bioinformatics. 2004 Oct 8;5:147. doi: 10.1186/1471-2105-5-147 (PMC528731; doi:10.1186/1471-2105-5-147)
Supplement: Additional File 2 — The original results of the above study (non-essential files are deleted to keep the file size under the limit set by BMC bioinformatics). [file 1471-2105-5-147-S2.bz2 › chilibotAdditionalFile2/dip05/263ID7973658E1060/html/IL2RG_JAK3.html]

 


 **IL2RG** and **JAK3** 
  
Found 74 abstracts in PubMed, retrieved 05.  
 

 What does Google say? 
 PDF only 
| .edu only 

---

**Interactive relationship** (e.g. stimulation, inhibition, etc)

**Neutral relationship**- Here we show that in normal lung fibroblasts IL 4 and IL 13 induce the expression of the gamma c  [ **IL2RG** ]  chain and its association with Janus kinase 3  [ **JAK3** ] , while lung myofibroblasts constitutively express a gamma c  [ **IL2RG** ]  chain displaying a limited association with  **JAK3** .  Ref: 12207328 Eur J Immunol, 2002
- Taken together, these results definitively demonstrate that IL 21 mediated signaling requires the gamma c  [ **IL2RG** ]  chain, and indicate that  **JAK3**  is an essential transducer of gamma c  [ **IL2RG** ]  dependent survival and or mitogenic signals induced by this cytokine.  Ref: 12093291 Biochemistry, 2002
- Nonreceptor tyrosine kinase  **Jak3** , which binds to intracellular domain of interleukin 2 receptor gamma  [ **IL2RG** ]  subunit IL 2Rgamma, plays an important role in IL 2 function.  Ref: 12136181 Sheng Wu Hua Xue Yu Sheng Wu Wu Li Xue Bao Shanghai < MedlineTA>Sheng Wu Hua Xue Yu Sheng Wu Wu Li Xue Bao Shanghai , 2002
- Retroviral mediated transduction of wild type gamma c  [ **IL2RG** ]  into XSCID  [ **IL2RG** ]  JT cells restored function to the IL 21R, as shown by IL 21 induced tyrosine phosphorylation of JAK1 and  **JAK3** , and downstream activation of STAT5, in JT gamma c  [ **IL2RG** ]  cells as well as BaF3 IL 21R alpha and primary splenic B cells.  Ref: 12093291 Biochemistry, 2002
- Evidence from peripheral T cells indicates that survival signals arising from the intermediate affinity IL 2R lacking CD25 do not require the activation of Janus kinase 3  [ **JAK3** ]   **Jak3**  but do require the presence of the membrane proximal region of the gamma C  [ **IL2RG** ]  chain.  Ref: 12121224 Immunol Cell Biol, 2002

**Non-interactive relationship** (e.g. studied together, co-existance, homology, etc.)

- In the latter cells, without exogenous cytokines, gamma c  [ **IL2RG** ]   **JAK3**  controls, through autocrine loops, tyrosine kinase 2 phosphorylation and the balance between functional and decoy high affinity receptors.  Ref: 12207328 Eur J Immunol, 2002
- The common gamma chain gamma c  [ **IL2RG** ]  is a required signaling component of the IL 21 receptor and supports IL 21 induced cell proliferation via  **JAK3** .  Ref: 12093291 Biochemistry, 2002
- Moreover, we demonstrate a functional requirement for both gamma c  [ **IL2RG** ]  and the gamma c  [ **IL2RG** ]  associated Janus family tyrosine kinase 3  **JAK3**  in IL 21 induced proliferation of pro B lymphoid cells engineered to express human IL 21R alpha BaF3 IL 21R alpha.  Ref: 12093291 Biochemistry, 2002
- These data underline the pivotal role of the gamma c  [ **IL2RG** ]  chain, CD40 CD40L,  **JAK3**  and IL 13 in the inflammatory like activation of lung myofibroblasts, the cell type restraint effects of IL 13 on these cells, and the potential usefulness of  **JAK3**  inhibitors in the treatment of asthma.  Ref: 12207328 Eur J Immunol, 2002
- In the disease, gamma c  [ **IL2RG** ]  is expressed, but its binding to  **Jak3**  is reduced.  Ref: 12126929 Mol Genet Metab, 2002
